# Fluoride Toothpaste Market Surveillance in the Dominican Republic: Quality Gaps and Policy Implications

**DOI:** 10.3290/j.ohpd.c_2783

**Published:** 2026-09-09

**Authors:** Ninoska Abreu-Placeres, Luis Eduardo Garrido, Frank Lippert, Kim Rud Ekstrand, George Eckert, Guillermo Tamayo-Cabeza, Stefania Martignon, Esperanza Angeles Martinez-Mier

**Affiliations:** a Ninoska Abreu-Placeres Researcher, Biomaterials and Dentistry Research Center (CIBO-UNIBE), Research and Innovation Department, Universidad Iberoamericana, Santo Domingo, Dominican Republic. Conceptualisation, data collection, data curation, data interpretation, visualisation, writing, review and editing of the original draft.; b Luis Eduardo Garrido Professor/Researcher, School of Psychology, Pontificia Universidad Católica Madre y Maestra, Santo Domingo, Dominican Republic. Formal analysis, data interpretation, visualisation, writing, review and editing of the original draft.; c Frank Lippert Professor, School of Dentistry, Oral Health Research Institute, Indiana University, Indianapolis, IN, USA. Laboratory analysis, Investigation, writing, review and editing of the original draft.; d Kim Rud Ekstrand Professor, Faculty of Health and Medical Sciences, Section of Cariology and Endodontics, University of Copenhagen, Copenhagen, Denmark. Funding acquisition, conceptualisation, writing, review and editing of the original draft.; e George Eckert Biostatistician, School of Medicine, Department of Biostatistics and Health Data Science, Indiana University, Indianapolis, IN, USA. Methodology, sample size calculation, writing review of the original draft.; f Guillermo Tamayo-Cabeza Research Assistant, Department of Dental Public Health and Dental Informatics, School of Dentistry, Indiana University, Indianapolis, IN, USA. Methodology, Statistical oversight, writing review of the original draft.; g Stefania Martignon Professor of Cariology, UNICA – Caries Research Unit, Research Department, Universidad El Bosque, Bogotá, Colombia. Conceptualisation, writing review of the original draft.; h Esperanza Angeles Martinez-Mier Professor and Chair, Department of Dental Public Health and Dental Informatics, School of Dentistry, Indiana University, Indianapolis, IN, USA. Conceptualisation, methodology, project administration, writing, review and editing of the original draft.

**Keywords:** fluorides, toothpastes, dental caries, Dominican Republic

## Abstract

**Purpose:**

The objective was to determine whether toothpastes marketed across the Dominican Republic provide fluoride levels that consistently meet internationally recommended benchmarks for caries prevention.

**Methods and Materials:**

A total of 22 toothpaste products representing 11 brands were sampled from four of the country’s most populated provinces. Six tubes per toothpaste were analysed using standardised fluoride measurement protocols. Total soluble fluoride (TSF) concentrations were evaluated against two internationally recognised benchmarks: ≥ 90% of labelled content and the 1000 ppm threshold for effective caries prevention. The proportion of toothpastes meeting these benchmarks was calculated, and Welch’s ANOVAs assessed regional variability in TSF.

**Results:**

Results showed that while 68.2% of toothpastes met both fluoride benchmarks, 31.8% failed to do so by containing insufficient TSF relative to labelled content, falling below the 1000-ppm threshold, or both. Toothpastes with sodium monofluorophosphate (SMFP) exhibited the highest non-soluble fluoride levels, averaging up to 44.1%. Significant variability in TSF concentrations was observed across provinces, with Greater Santo Domingo consistently showing lower TSF levels than other regions. Locally manufactured toothpastes and products with incompatible abrasives demonstrated the greatest fluoride losses.

**Conclusions:**

Our findings provide market-wide surveillance evidence from a low- and middle-income country directly supporting WHO’s Global Oral Health Action Plan (2023–2030). They highlight quality assurance gaps in fluoride toothpaste that undermine population-level caries prevention. These results underscore the global need for routine market monitoring, ISO-concordant standards, and fiscal policies to ensure effective, affordable fluoride toothpaste is reliably available worldwide.

Fluoride toothpaste is a vital preventive measure against dental caries, a disease that remains a major global burden despite decades of prevention efforts.^4,6
^ The decline in caries prevalence in many countries has been strongly linked to the widespread use of fluoride toothpaste, establishing it as a cornerstone of modern oral health strategies.^24^ Beyond the mechanical removal of biofilm, toothbrushing delivers fluoride to the oral environment, promoting enamel remineralisation and reducing demineralisation.^10^ This combination of chemical and mechanical actions optimises caries prevention by emphasising the importance of daily fluoride exposure.^1^ Recognising its significance, the World Health Organization (WHO) has included fluoride toothpaste in its Model List of Essential Medicines, emphasising its essential role in preventive care and global health policy.^27,30
^ Given its widespread use and critical role in prevention, ensuring proper formulation and adequate fluoride levels in commercial toothpastes remains a key concern for public health and regulatory agencies worldwide.

This challenge is particularly acute in low- and middle-income countries (LMICs), where fluoride toothpaste often represents the primary, and sometimes only, source of population-level fluoride exposure due to limited access to public health fluoridation programmes.^21,27
^ The WHO’s Global Oral Health Action Plan (2023–2030) specifically emphasises the need for routine market surveillance for fluoride toothpaste, recognising that quality assurance gaps in these products can undermine population-level prevention efforts globally.^29^ Despite this recognised need, systematic market surveillance data from LMICs remains limited, creating knowledge gaps that hinder evidence-based policy development and delay regulatory harmonisation across regions facing similar public health challenges.

The most commonly used fluoride compounds in toothpaste include sodium fluoride (NaF), sodium monofluorophosphate (SMFP; Na_2_FPO_3_), amine fluoride (AmF), and stannous fluoride (SnF_2_), each with distinct properties that influence bioavailability and remineralisation efficacy.^1,17
^ To maximise benefits and reduce the risk of excessive exposure, regulatory agencies have issued guidelines on fluoride concentrations in toothpastes. Institutions such as the US Food and Drug Administration (FDA), the European Union, and the World Health Organization (WHO) generally recommend levels between 1000 and 1,500 ppm to balance efficacy and safety.^12,25,30
^ Two international benchmarks are especially relevant in this context: first, toothpastes should contain at least 1000 ppm fluoride to provide clinically meaningful caries-preventive benefits^28^; and second, the concentration of soluble fluoride should reach at least 90% of the labelled claim to ensure adequate bioavailability.^2^


Although the fluoride concentration in toothpaste is a major factor in dental caries prevention, its bioavailability ultimately determines its anticaries potential. Measurement of total fluoride (TF) alone does not guarantee efficacy, because chemical interactions in the formulation can limit the amount of fluoride available for enamel remineralisation.^24^ Factors such as calcium-based abrasives or instability in the toothpaste mixture can reduce free, ionisable fluoride and weaken its protective effect.^17,19
^ Consequently, both regulatory agencies and researchers emphasise the importance of analytical methods that verify not only labelled fluoride content but also the fraction that is bioavailable during brushing.^20,27
^ Studies worldwide have reported discrepancies between labelled and measured fluoride, with some products containing less bioavailable fluoride than expected, raising concerns about product quality and monitoring.^19^


Despite these well-documented discrepancies between labelled and bioavailable fluoride, no published research has assessed whether fluoride concentrations in toothpastes sold in the Dominican Republic align with manufacturer claims or meet internationally recommended thresholds. Furthermore, it remains unclear whether fluoride content varies geographically within the Dominican Republic. Thus, this study aimed to determine whether toothpastes marketed in the Dominican Republic provide adequate fluoride levels to meet internationally recommended benchmarks for caries prevention and to assess the consistency of fluoride availability across major provinces.

## Methods and Materials

This *in vitro* study did not require ethical approval as it did not involve human participants, animal subjects, or identifiable personal data.

### Sample Size Calculation

To determine the required sample size for accurately estimating the fluoride content of each toothpaste type, we employed the standard error of the mean formula^3^:



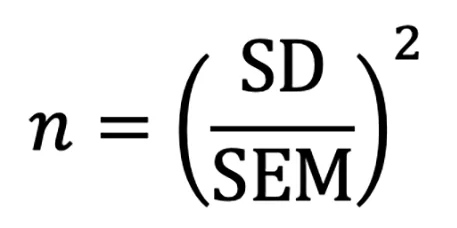



where n denotes the number of tubes analysed per toothpaste type, SD is the estimated standard deviation of fluoride concentrations among tubes of that type, and SEM is the standard error of the mean fluoride concentration.

A pilot study was conducted to obtain preliminary estimates of SD. Seven toothpaste brands available in supermarkets in Santo Domingo were selected, including three formulations marketed for children and four for adults. Four products contained sodium fluoride (NaF): three with a labelled concentration of 1100 ppm and one with 1450 ppm. Two contained sodium monofluorophosphate (SMFP): one with 1000 ppm and one with 1450 ppm. One contained stannous fluoride (SnF_2_) with a labelled fluoride concentration of 1000 ppm. Two tubes of each product type from different lots were purchased, resulting in 14 samples. For analysis, toothpastes with the same fluoride compound and concentration were grouped to calculate SD for TF and TSF. For example, the three NaF toothpastes labelled with 1100 ppm were treated as a single group. Because the required sample size is directly proportional to the SD, the largest value from the pilot study (12.48) was used to calculate the most conservative estimate of n, ensuring adequate precision across all toothpaste types.

To establish the target SEM, we considered the international benchmark that TSF should represent at least 90% of the labelled concentration. This threshold corresponds to an allowable deviation of up to 100 ppm for a product labelled at 1000 ppm. To achieve higher precision, we selected a confidence interval half-width of ± 10 ppm (1% of the labelled concentration) as our target. Using a z value of 1.96 for a 95% confidence interval, the SEM was calculated as 10 ppm/1.96 = 5.10. Based on this value, the required number of tubes per toothpaste type was determined as follows:



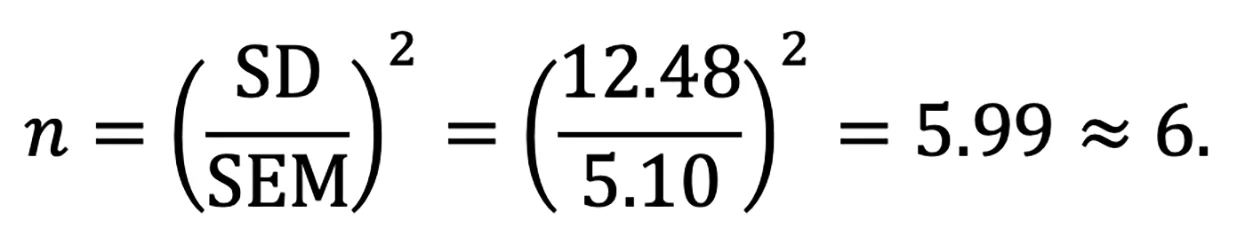



### Sample Purchasing Strategy

**Fig 1 Fig1:**
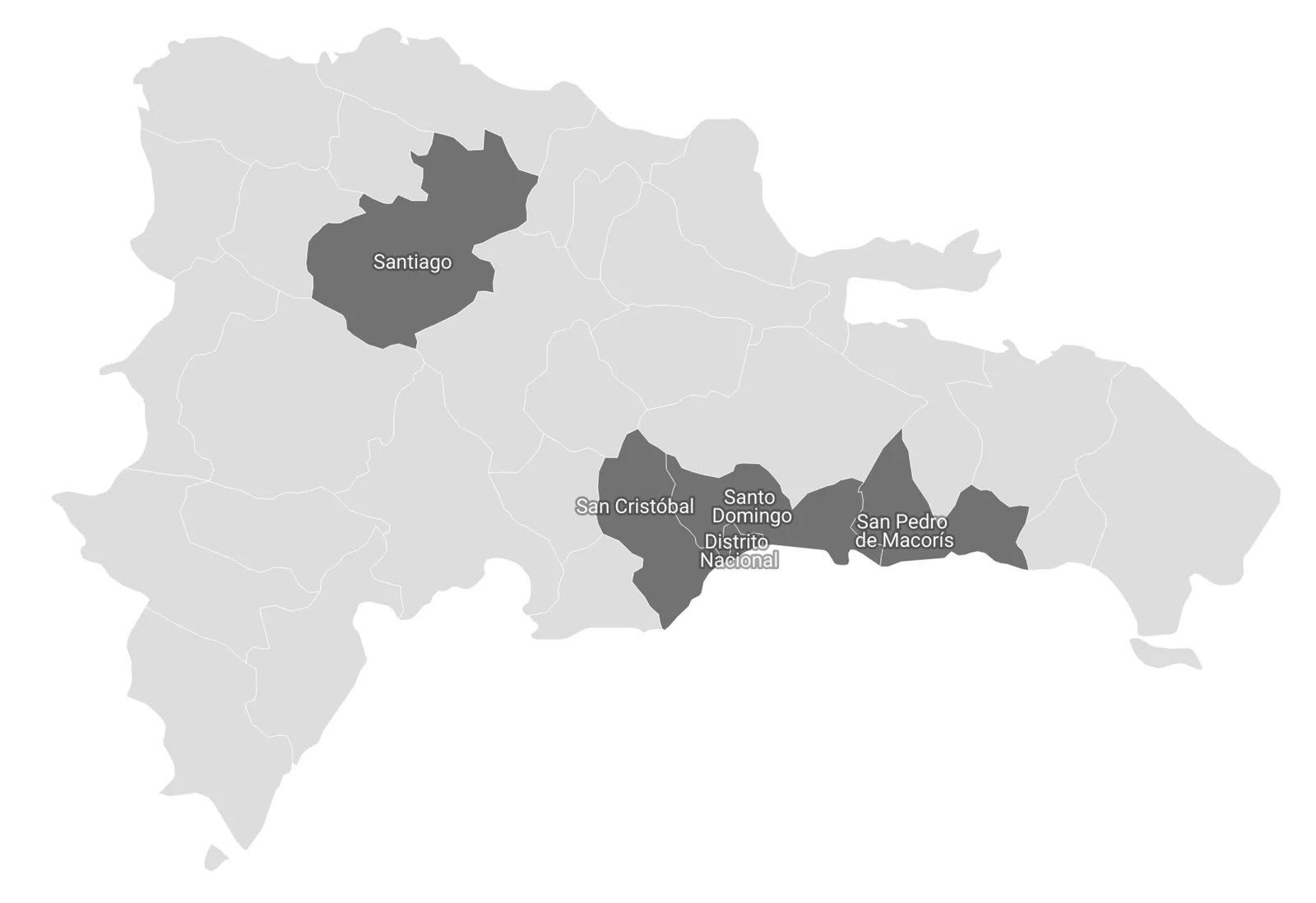
Map of provinces sampled in the Dominican Republic.

To capture both geographic and commercial variability, toothpastes were purchased from four of the most populous provinces in the Dominican Republic: Greater Santo Domingo (GSD) and San Pedro de Macorís (SPM) in the Southeast region, San Cristóbal (SC) in the Southwest, and Santiago (STGO) in the North (Fig 1). Within each province, retail outlets were selected to reflect the diversity of the local supply chain and consumer purchasing behaviour. These included superstores (29.2% of samples), large chain supermarkets (17.5%), small neighbourhood stores locally known as colmados (6.7%), pharmacies (27.5%), premium supermarkets (16.7%), and, when available, wholesale distributors (2.5%).

The Dominican toothpaste market consists of a wide variety of products, primarily from multinational brands, along with two local brands (one manufactured domestically and the other imported). In the absence of a publicly available database listing the brands and product variants sold in the country, the research team obtained a list of toothpaste brands and product lines from a private marketing agency. This list was subsequently verified and expanded through an on-site market survey, which involved visiting the retail outlets described above to document available brands, product variants, fluoride compounds, and intended uses (e.g. anticavity, tartar control, whitening, or paediatric formulations). When accessible, official brand websites were also reviewed; however, only two multinational companies provided country-specific information online. Based on the combined information from the marketing agency, field survey, and online sources, a sampling plan was developed to achieve comprehensive representation of the national toothpaste market.

The final selection comprised 22 distinct toothpaste types: 6 that were commonly available across all four provinces and 16 that represented the largest market, GSD. For each type, six samples were purchased from different stores within each province where it was available, ensuring each came from a separate retail source. Tubes were also chosen from different production batches. This approach resulted in a total of 240 samples (6 [types] × 4 [provinces] × 6 [samples] + 16 [types] × 1 [province] × 6 [samples]). Details of the selected toothpaste types are provided in Table 1.

**Table 1 Table1:** Summary of sampled toothpaste specifications

Commercial brand			Fluoride			
	Toothpaste	Fluoride	concentration	Abrasive agent	Toothpaste	Provinces
	codea	compound	declared (ppm)	declared	labelling	sampled
Colgate Junior Barbie	A1	NaF	1100	Hydrated silica	For children	All
Colgate Triple Acción	A2	NaF	1450	Hydrated silica	Anticavity	All
Colgate Anticavity	A3	SMFP	1450	Dicalcium phosphate	Anticavity	All
Colgate Natural Extracts Cítricos	A4	NaF	1000	Hydrated silica	Anticavity	GSD
Colgate Smiles Minions	A5	NaF	1100	Hydrated silica	For children	GSD
Colgate Total Clean Mint	A6	NaF	1450	Hydrated silica	Total protection	GSD
Oral B Complete	B1	NaF	1450	Silica	Total protection	All
Oral B 3D Whit B Fresh	B2	NaF	1100	Hydrated silica	Whitening	GSD
Oral B Kids C/D Mickey	B3	NaF	1100	Hydrated silica	For children	GSD
Oral B Stages Frozen	B4	NaF	1100	Hydrated silica	For children	GSD
Crest Tartar Control	C1	NaF	1100	Hydrated silica	Tartar control	All
Crest Gum Detoxify	C2	SnF_2_	1000	Hydrated silica	Gums	All
Oddent Fluor Infantil	D1	NaF	500	Not declared	For children	GSD
Oddent Bifluor	D2	SMFP	1487	Not declared	Anticavity	GSD
Vitis Encías	E1	NaF	1450	Silica	Gums	GSD
Vitis Blanqueadora	E2	SMFP	1450	Silica	Whitening	GSD
A&H Comp Care Whitening	F	NaF	1100	Hydrated silica	Whitening	GSD
Sensodyne Repair & Pro	G	NaF	1426	Hydrated silica	Repair	GSD
Gingi Lacer Fluor	H	SMFP	1500	Hydrated silica	Gums	GSD
Lacer Antiplaca, Anticaries	I	SMFP	2500	Calcium carbonate	Cavity and plaque protection	GSD
Pyodent	J^b^	NaF	1100	Calcium carbonate	Gums	GSD
Crema Dental Bravo	K	NaF	1450	Hydrated silica	Total protection	GSD
*Note*: NaF = sodium fluoride; SMFP = sodium monofluorophosphate; SnF_2_ = stannous fluoride; GSD = Greater Santo Domingo. All toothpastes were within expiration date.						
^a^ Toothpastes sharing the same letter are from the same brand.						
^b^ Locally manufactured.						


### Fluoride Measurement Protocol

#### Sample preparation and handling

After collection, toothpaste samples were prepared, coded, and shipped to the Oral Health Research Institute at the Indiana University School of Dentistry. Upon receipt, analyses were conducted in accordance with US Food and Drug Administration (FDA) protocols for fluoride toothpaste analysis.^26^ For quality control, 10% of the samples were reanalysed.

#### General analytical procedure

All fluoride analyses were performed according to the procedures described in the FDA Monograph, with minor adaptations where required. Fresh toothpaste samples were weighed to the nearest 0.001 g and mixed with deionised water at the specified dilution. Slurries were stirred with a laboratory mixer and stir bar and centrifuged when required. Aliquots were combined with Total Ionic Strength Adjustment Buffer (TISAB) II (or TISAB IV for Product A). Fluoride concentrations were determined using a fluoride ion-specific electrode and an ion analyser or pH/ISE meter. For each test, a standard fluoride calibration curve was prepared and used to calculate fluoride content.

##### Test #1: Total fluoride (NaF and SnF_2_)

Total fluoride was determined following the procedure for Test #1 in the FDA Monograph, with minor adaptations. Samples of 0.250 g were diluted 1:100 with 25 ml deionised water. Each slurry was mixed for 1 min with a stand mixer and then stirred for an additional 19 min at 300 rpm, for a total mixing time of 20 min. A 1.0 ml aliquot of each slurry was mixed with 1.0 ml TISAB II (or TISAB IV for Product A).

##### Test #29: Total soluble fluoride (NaF and SnF_2_)

Total soluble fluoride was determined following the procedure for Test #29 in the FDA Monograph, with minor adaptations. Samples of approximately 2.5 g were diluted 1:10 with 25 ml deionised water. Each slurry was mixed for 1 min with a non-aerating mixer and then stirred for an additional 4 min at 300 rpm, for a total mixing time of 5 min. Slurries were centrifuged at 11,000 g for 8 min. A 1.0 ml aliquot of each supernatant was mixed with 1.0 ml TISAB II.

##### Test #3: Total fluoride (SMFP)

Total fluoride for SMFP toothpastes was determined following the procedure for Test #3 in the FDA Monograph, with minor adaptations. Petri dish covers were prepared by adding 0.3 ml of 0.25 N ethanolic sodium hydroxide and allowing the alcohol to evaporate under vacuum. Samples of 0.250 g were diluted 1:100 with 25.0 ml deionised water using a non-aerating mixer. A 2.0 ml aliquot of each slurry was transferred to the bottom of a prepared Petri dish. Subsequently, 4.0 ml of 70% HClO_4_ were added, and the sodium hydroxide-coated lid was immediately placed on each dish. Dishes were incubated in an oven at 60°C overnight. After removal, lids were washed multiple times with deionised water, and the washings were collected in a 25 ml volumetric flask and brought to volume with deionised water. This solution was diluted 1:1 with TISAB II before analysis.

##### Test #16: Total soluble fluoride (SMFP)

Total soluble fluoride for SMFP dentifrices was determined following the procedure for Test #16 in the FDA Monograph, with minor adaptations. Petri dish covers were prepared as described for Test #3. Samples of 2.5 g were diluted 1:10 with 25.0 ml deionised water using a non-aerating mixer. Slurries were centrifuged at 11,000 g for 8 min. A 2.0 ml aliquot of each supernatant was transferred to the bottom of a prepared Petri dish. Subsequently, 4.0 ml of 70% HClO₄ were added, and the sodium hydroxide-coated lid was immediately placed on each dish. Dishes were incubated in an oven at 60°C overnight. After removal, lids were washed multiple times with deionised water, and washings were collected in a 25 ml volumetric flask and brought to volume with deionised water. This solution was diluted 1:1 with TISAB II before analysis.

### Statistical Analyses

Due to the small sample size for each toothpaste type (n = 6), confidence intervals for the mean fluoride content were calculated using the critical value from a t distribution with n − 1 degrees of freedom.^16^ For a sample size of six, the critical value for a 95% confidence interval is 2.57. Accordingly, 95% confidence intervals (95% CIs) were constructed as M ± 2.57 (SD/√n), where M is the mean and SD is the standard deviation. A toothpaste was considered to fall below a fluoride benchmark when the entire 95% confidence interval (CI) for TSF lay below that benchmark (i.e. the CI upper bound < benchmark). The two benchmarks considered were (1) 90% of the labelled concentration,^2^ or (2) the 1000 ppm threshold recognised as the minimum effective level for caries prevention.^28^ The variability of TSF across provinces was assessed using Welch’s analysis of variance (ANOVA), which does not assume equal variances across groups,^11^ followed by Games–Howell post hoc comparisons to identify significant pairwise differences. Data management, descriptive analyses, and Welch’s ANOVAs were performed using IBM SPSS Statistics (version 25).

## Results

### Fluoride Content of Toothpastes Sampled in the Four Selected Provinces

Toothpastes sampled in GSD, SC, SPM, and STGO generally showed TF levels that aligned with their labelled content (Table 2). All products met the minimum 1000 ppm threshold for TF, except for C2, which fell below this level in all provinces. For TSF, five out of six products met both the 90% benchmark relative to the labelled concentration and the 1000 ppm threshold across all provinces. Only toothpaste C2 failed to meet either benchmark in all locations. Among formulations that met the benchmarks, A3 showed the highest proportion of non-soluble fluoride, ranging from 7.3% to 10.3%.

**Table 2 Table2:** Fluoride content of toothpastes sampled in the four selected provinces

Toothpaste code^c^	Province	Fluoride compound	Fluoride declared (ppm)	90% of labelled fluoride (ppm)	Total fluoride (ppm)			Total soluble fluoride (ppm)			Non- soluble%
					M	SD	95% CI	M	SD	95% CI	
A1	GSD	NaF	1100	990	1149.3	2.31	1146.9–1151.7	1088.1	7.68	1080.0–1096.1	5.3
	SC				1108.9	4.38	1104.3–1113.5	1112.4	3.34	1108.9–1115.9	–0.3^d^
	SPM				1102.3	5.35	1096.6–1107.9	1105.1	4.27	1100.6–1109.6	–0.3^d^
	STGO				1105.2	2.24	1102.8–1107.5	1110.5	9.22	1100.8–1120.1	–0.5^d^
A2	GSD	NaF	1450	1305	1454.8	4.20	1450.4–1459.2	1396.2	9.35	1386.4–1406.0	4.0
	SC				1424.5	14.98	1408.8–1440.2	1430.2	6.63	1423.2–1437.1	–0.4^d^
	SPM				1384.8	15.00	1369.1–1400.5	1407.2	11.94	1394.7–1419.7	–1.6^d^
	STGO				1408.1	17.44	1389.8–1426.4	1421.9	9.29	1412.2–1431.7	–1.0^d^
A3	GSD	SMFP	1450	1305	1458.9	9.71	1448.7–1469.1	1308.5	15.75	1292.0–1325.1	10.3
	SC				1431.8	13.87	1417.2–1446.3	1304.4	21.66	1281.7–1327.1	8.9
	SPM				1421.8	20.48	1400.3–1443.3	1318.5	15.37	1302.4–1334.6	7.3
	STGO				1404.5	12.28	1391.6–1417.3	1301.8	17.94	1283.0–1320.7	7.3
B1	GSD	NaF	1450	1305	1465.8	4.55	1461.1–1470.6	1398.5	2.85	1395.5–1401.5	4.6
	SC				1445.6	6.76	1438.5–1452.7	1402.8	4.46	1398.1–1407.5	3.0
	SPM				1448.5	7.88	1440.2–1456.8	1408.9	5.56	1403.1–1414.8	2.7
	STGO				1433.5	4.14	1429.2–1437.8	1417.4	4.90	1412.3–1422.6	1.1
C1	GSD	NaF	1100	990	1113.5	5.84	1107.4–1119.6	1062.7	15.28	1046.7–1078.7	4.6
	SC				1106.1	3.45	1102.4–1109.7	1070.3	3.46	1066.6–1073.9	3.2
	SPM				1109.4	4.87	1104.3–1114.5	1075.7	6.65	1068.7–1082.7	3.0
	STGO				1106.1	6.48	1099.3–1112.9	1073.7	6.19	1067.2–1080.2	2.9
C2	GSD	SnF_2_	1000	900	966.4^b^	3.13	963.1–969.7	788.8^ab^	15.23	772.9–804.8	18.4
	SC				927.3^b^	11.90	914.8–939.8	740.4^ab^	10.56	729.3–751.5	20.2
	SPM				949.0^b^	12.88	935.5–962.5	773.4^ab^	16.86	755.7–791.1	18.5
	STGO				960.6^b^	15.56	944.3–976.9	791.7^ab^	21.02	769.6–813.7	17.6
*Note*: GSD = Greater Santo Domingo; SC = San Cristóbal; SPM = San Pedro de Macorís; STGO = Santiago; CI = confidence interval; NaF = sodium fluoride; SMFP = sodium monofluorophosphate; SnF_2_ = stannous fluoride. n = 6 for all toothpaste-province combinations.											
^a^ Fails to meet the 90% of labelled fluoride benchmark.											
^b^ Fails to meet the 1000 ppm minimum threshold benchmark.											
^c^ Toothpastes sharing the same letter are from the same brand.											
^d^ Small negative values reflect measurement error and should be interpreted as 0.0%.											


Welch’s ANOVAs revealed significant differences in TSF across provinces for four toothpastes: A1 (F_3,10.49_ = 15.94, *p* < 0.001), A2 (F_3,10.88_ = 17.45, *p* < 0.001), B1 (F_3,10.71_ = 21.60, *p* < 0.001), and C2 (F_3,10.77_ = 17.26, *p* < 0.001). Games–Howell post hoc tests indicated that A1 showed significant pairwise differences for GSD-SC (*p* = 0.001), GSD-SPM (*p* = 0.007), GSD-STGO (*p* = 0.005), and SC-SPM (*p* < 0.036), with a maximum mean difference of 24.3 ppm for GSD-SPM. Toothpaste A2 exhibited significant differences for GSD-SC (*p* < 0.001), GSD-STGO (*p* = 0.003), and SC-SPM (*p* = 0.015), the largest being 33.95 ppm for GSD-SC. For B1, significant differences were found in GSD-SPM (*p* = 0.017), GSD-STGO (*p* < 0.001), and SC-STGO (*p* = 0.001), with a maximum mean difference of 18.98 ppm for GSD-STGO. The final toothpaste with significant provincial variation, C2, showed differences for GSD-SC (*p* = 0.001), SC-SPM (*p* = 0.014), and SC-STGO (*p* = 0.004), with the largest mean difference of 51.29 ppm for SC-STGO.

**Fig 2 Fig2:**
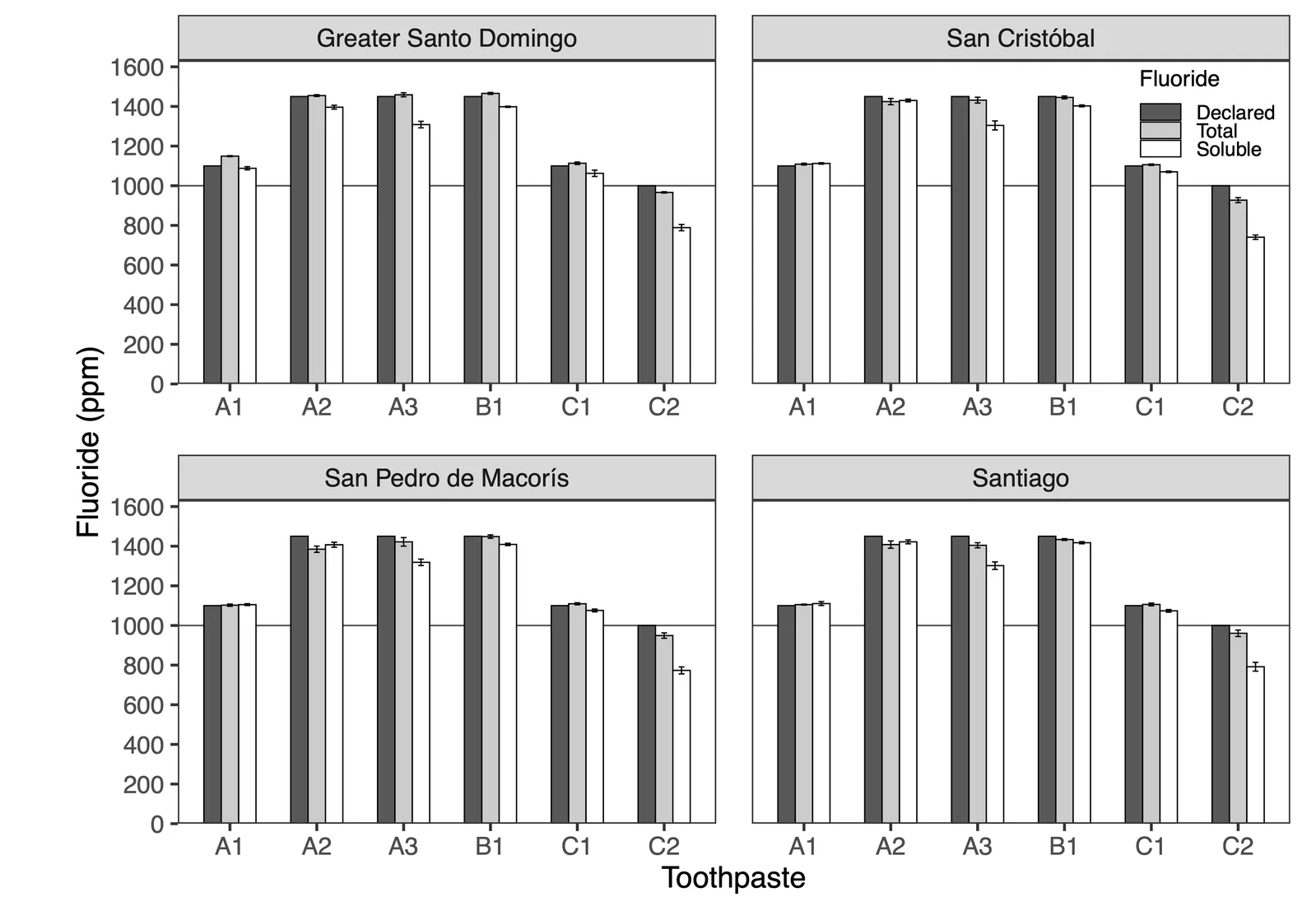
Declared and Measured Fluoride Content of Toothpastes Sampled in the Four Selected Provinces. Note: Solid horizontal lines indicate the 1000 ppm fluoride threshold commonly used as a benchmark for caries prevention efficacy. Error bars denote the 95% confidence intervals for the measured fluoride means.

Notably, in seven of the eight significant pairwise differences involving GSD, this province had the lowest TSF levels. This pattern is consistent with Table 2, where for five of the six toothpastes (all except C2), samples from GSD showed the highest percentages of non-soluble fluoride. The two toothpastes that did not show significant provincial differences were A3 (F_3,11.04_ = 1.05, *p* = 0.411) and C1 (F_3,10.25_ = 1.75, *p* = 0.219). Figure 2 provides a graphical comparison of the declared and measured fluoride concentrations for each toothpaste across the four provinces, including their corresponding 95% CIs.

### Fluoride Content of Toothpastes Sampled Only in GSD

**Fig 3 Fig3:**
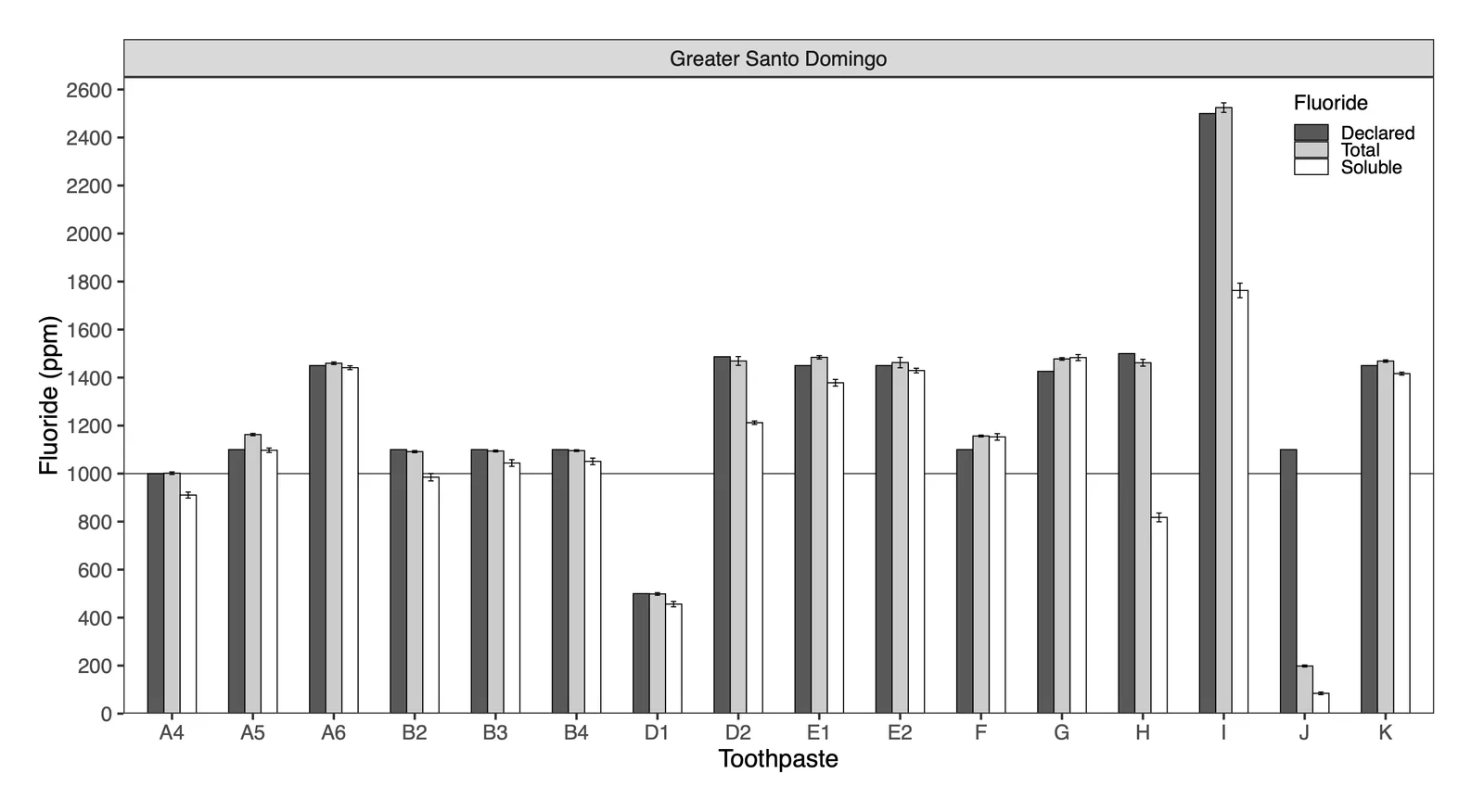
Declared and measured fluoride content of toothpastes sampled only in the Greater Santo Domingo Province.

Among the toothpastes sampled exclusively in GSD, the largest market in the country, 62.5% met both international benchmarks: the minimum fluoride concentration of 1000 ppm and at least 90% of the labelled content (A5, A6, B2, B3, B4, E1, E2, F, G, and K; Table 3). An additional 25.0% met only one of these benchmarks. Two products fell below the minimum fluoride threshold (A4 and D1), while two others did not reach the 90% benchmark relative to their labelled content (D2 and I). Only two toothpastes failed to meet either benchmark, both showing particularly low levels of TF and TSF (H and J). Formulation J exhibited markedly deficient fluoride content, with 95% CI upper bounds of only 202.6 ppm for TF and 90.0 ppm for TSF. High proportions of non-soluble fluoride were observed in four products—three containing SMFP (H, I, and D2) and one with NaF (J)—ranging from 17.5% to 57.4%. Figure 3 displays a grouped bar chart comparing declared fluoride, TF, and TSF for each toothpaste sampled exclusively in GSD, along with their corresponding 95% CIs.

**Table 3 Table3:** Fluoride content of toothpastes sampled only in the Greater Santo Domingo Province

Toothpaste code^c^	Fluoride compound	Fluoride declared (ppm)	90% of labelled fluoride (ppm)	Total fluoride (ppm)			Total soluble fluoride (ppm)			Non- soluble%
				M	SD	95% C.I.	M	SD	95% CI	
A4	NaF	1000	900	1001.7	5.11	996.3–1007.0	910.6^b^	11.92	898.1–923.1	9.1
A5	NaF	1100	990	1162.6	4.66	1157.7–1167.5	1097.3	8.78	1088.1–1106.5	5.6
A6	NaF	1450	1305	1459.7	5.01	1454.5–1465.0	1441.1	7.70	1433.1–1449.2	1.3
B2	NaF	1100	990	1091.5	4.31	1087.0–1096.0	985.0	14.77	969.5–1000.5	9.8
B3	NaF	1100	990	1094.2	3.75	1090.3–1098.1	1044.1	13.24	1030.2–1058.0	4.6
B4	NaF	1100	990	1095.7	3.43	1092.1–1099.3	1050.9	13.12	1037.1–1064.6	4.1
D1	NaF	500	450	498.5^b^	5.21	493.1–504.0	456.2^b^	10.80	444.8–467.5	8.5
D2	SMFP	1487	1338	1469.2	17.49	1450.8–1487.6	1212.1^a^	6.75	1205.0–1219.2	17.5
E1	NaF	1450	1305	1484.5	6.75	1477.4–1491.6	1378.4	13.34	1364.4–1392.4	7.1
E2	SMFP	1450	1305	1462.6	20.78	1440.8–1484.4	1429.3	9.22	1419.7–1439.0	2.3
F	NaF	1100	990	1156.7	3.63	1152.9–1160.5	1152.8	13.03	1139.1–1166.5	0.3
G	NaF	1426	1283	1477.8	5.11	1472.4–1483.1	1483.3	11.95	1470.8–1495.9	–0.4^d^
H	SMFP	1500	1350	1461.8	13.59	1447.6–1476.1	817.6^ab^	17.41	799.4–835.9	44.1
I	SMFP	2500	2250	2524.7	18.95	2504.8–2544.6	1762.9^a^	29.03	1732.4–1793.3	30.2
J^e^	NaF	1100	990	198.5^ab^	3.91	194.4–202.6	84.6^ab^	5.19	79.1–90.0	57.4
K	NaF	1450	1305	1468.9	4.65	1464.0–1473.8	1416.5	5.78	1410.4–1422.5	3.6
*Note: *CI = confidence interval; NaF = sodium fluoride; SMFP = sodium monofluorophosphate; SnF_2_ = stannous fluoride. n = 6 for all toothpastes.										
^a^ Fails to meet the 90% of labelled fluoride benchmark.										
^b^ Fails to meet the 1000 ppm minimum threshold benchmark.										
^c^ Toothpastes sharing the same letter are from the same brand.										
^d^ Small negative values reflect measurement error and should be interpreted as 0.0%.										
^e^ Locally manufactured.										


### Overall Benchmark Analysis of Fluoride Content

The fluoride content of 22 toothpastes was evaluated in the present study. Of these, five (22.7%; C2, D2, H, I, and J) fell below the 90% benchmark relative to their labelled concentration. Likewise, five (22.7%; A4, C2, D1, H, and J) did not meet the 1000 ppm threshold recognised as the minimum level for effective caries prevention. In total, seven toothpastes (31.8%) failed to meet at least one of these two benchmarks. Benchmark attainment varied by fluoride compound. Among NaF formulations (n = 16), 93.8% met the 90% benchmark and 81.3% met the minimum 1000 ppm threshold. For SMFP toothpastes (n = 5), 60.0% met the 90% benchmark and 80.0% met the minimum threshold. The single SnF_2_ toothpaste fell below both benchmarks.

## Discussion

Caries prevention in many low- and middle-income countries (LMICs), such as the Dominican Republic, depends primarily on fluoride toothpaste, making the quality and reliability of these products a matter of global public health concern. Recognising this is the situation of many LMICs, the WHO recommends twice-daily use of toothpaste containing 1000–1500 ppm fluoride and, through its Global Oral Health Action Plan 2023–2030, urges Member States to strengthen availability, affordability, and ISO 11609–aligned quality and labelling standards. Our market-wide testing from the Dominican Republic provides concrete surveillance evidence from an LMIC setting that speaks directly to these priorities, documenting quality assurance gaps that may undermine population-level prevention. These findings also complement WHO’s recent elevation of dental preparations, including fluoride toothpaste, within the Model Lists of Essential Medicines,^30^ a policy shift designed to facilitate regulation, procurement, and pricing measures. Together, our findings and these global policy developments argue for the need to implement routine national market surveillance, enforce ISO-concordant standards and labelling, and adopt fiscal policies (e.g. reductions in consumption taxes) to ensure that fluoride toothpaste of adequate concentration is affordable and reliably available. Affordability has been identified as a major barrier to effective fluoride toothpaste coverage globally, including in LMIC settings, reinforcing the need for policies that support access alongside quality assurance and surveillance.^14,15
^


This study presents the first comprehensive evaluation of fluoride content in commercially available toothpastes in the Dominican Republic. A total of 22 products representing 11 brands were purchased from four of the country’s most populous provinces. Sodium fluoride (NaF) was the most common compound, present in 72.7% of products, followed by sodium monofluorophosphate (SMFP) in 22.7% and stannous fluoride (SnF_2_) in 4.5%, reflecting trends observed in other Latin American markets.^13,23
^ Total fluoride (TF) and total soluble fluoride (TSF) concentrations were measured and compared with labelled claims using a 90% benchmark relative to declared content,^2^ as well as the internationally recommended minimum of 1000 ppm TSF required for effective caries prevention.^8,19,28
^ Overall, 68.2% of toothpastes met both benchmarks, while nearly one-third (31.8%) fell short of at least one by containing less than 1000 ppm soluble fluoride, failing to reach 90% of their labelled concentration, or both. These results are congruent with several studies that have assessed TF and TSF concentrations in commercially available toothpastes in multiple countries, consistently identifying discrepancies in fluoride bioavailability relative to established benchmarks. Investigations conducted in Belgium, Brazil, Chile, Indonesia, Peru, and Uruguay reported significant mismatches between labelled and measured fluoride concentrations, raising concerns regarding product efficacy in preventing dental caries.^5,7,9,13,17,18,22,23
^


A common methodological limitation of prior studies, however, is their reliance on mean fluoride concentrations derived from minimal sampling (often only two or three tubes per brand), without employing confidence intervals or other inferential statistical methods. Such an approach can obscure within-brand variability and potentially lead to misclassification of whether products meet benchmarks. In contrast, the larger sample sizes in this study enabled inferential statistical analysis with adequate power, providing a more robust and generalisable assessment of whether toothpastes met established fluoride benchmarks. These results have important public health implications, as potable water is not fluoridated in the country and toothpaste represents the main source of fluoride exposure, a situation shared by many LMICs.

A closer examination of specific fluoride compounds and regional patterns revealed important factors underlying benchmark outcomes. Toothpastes containing sodium monofluorophosphate (SMFP) displayed higher levels of non-soluble fluoride, with some products averaging up to 44.1%. This aligns with previous research showing that SMFP formulations, particularly when combined with calcium-based abrasives, can undergo hydrolysis during storage, leading to the formation of inactive fluoride complexes and reduced bioavailability.^8,23
^ In addition, statistically significant differences in TSF concentrations were observed among provinces, with the largest difference of 51.29 ppm between San Cristóbal and Santiago. This variation could meaningfully influence whether formulations close to either benchmark, the 1000 ppm minimum or the 90% of labelled content, are classified as meeting or falling short.

Toothpastes collected from GSD, the most populous region, consistently showed the highest percentages of non-soluble fluoride across provinces. One possible explanation is that retail outlets in this urban area often maintain larger inventories with slower turnover, potentially resulting in longer storage times that affect fluoride stability. Considered collectively, these findings highlight potential challenges related to quality control, storage, and distribution practices, factors identified in other countries as influential on fluoride stability and bioavailability.^19,23
^


### Limitations

Our study has several limitations that should be considered when interpreting the results. First, toothpaste samples were primarily acquired from major urban centres, which may not reflect conditions in rural areas where supply chains, storage, and product turnover could differ substantially, potentially influencing whether products meet fluoride benchmarks.^19^ Second, although our analysis provided a snapshot of fluoride levels at the time of purchase, we did not evaluate fluoride stability across the full shelf life of the products. Previous studies have shown that soluble fluoride can decline markedly over time, particularly in formulations containing monofluorophosphate or calcium-based abrasives.^8,18
^ Future surveillance studies should incorporate repeated measurements across product shelf life, including laboratory ageing when feasible, to quantify changes in soluble fluoride, particularly for formulations susceptible to hydrolysis or interactions with calcium-based abrasives.^18^ Finally, we were unable to sample all toothpaste types in every province, primarily because some products were unavailable in certain locations and because funding limited broader sampling. These factors reduced full provincial representation and may have affected the generalizability of our results. Despite these limitations, our findings provide important insights into the extent to which toothpastes marketed in the Dominican Republic meet international fluoride benchmarks and highlight areas for future research.

### Practical Implications

Across Latin America and the Caribbean, regulations aligned with WHO recommendations and national standards are urgently needed to ensure that toothpaste formulations consistently meet benchmarks for effective caries prevention.^21,29
^ In the Dominican Republic, current regulatory documents specify only maximum fluoride concentrations without defining minimum levels of bioavailable fluoride, reflecting a regulatory gap that could compromise public oral health. Although a 2022 resolution from the General Department of Medicines, Foods, and Health Products (DIGEMAPS) mandates alignment with European standards for cosmetic and personal hygiene products, explicit and enforceable guidelines specific to toothpaste formulations remain undefined. The presence on the market of a toothpaste labelled at 2500 ppm fluoride, which could be obtained in pharmacies without a prescription despite requiring one for sale, further highlights the need for clearer regulatory classification, dispensing requirements, and stronger enforcement of toothpaste-specific standards.

Our findings reveal specific formulation challenges that compromise fluoride bioavailability in the Dominican Republic market. The product with the greatest fluoride loss was locally manufactured and contained an abrasive incompatible with its fluoride compound, highlighting formulation issues that can reduce efficacy. Furthermore, toothpastes formulated with sodium monofluorophosphate (SMFP) paired with calcium-based abrasives (common among lower-priced products) are prone to reduced soluble fluoride due to hydrolysis during storage.^17,23
^ The widespread use of these chemically unstable formulations risks exposing vulnerable populations to fluoride levels below recommended benchmarks, potentially undermining caries prevention efforts in groups that can least afford dental treatment.^21^


### Acknowledgements

The authors would like to thank Chris Buckley for conducting the laboratory analyses, as well as Eva Reinwald and Paula Yunes for their valuable support throughout this project. We are also grateful to the University of Copenhagen for providing financial support that made this work possible. The authors acknowledge the use of artificial intelligence tools (ChatGPT, OpenAI), specifically the GPT-5o, to assist with grammar and language refinement aimed at enhancing clarity, coherence, and readability of the manuscript.

## References
